# Ventilation with lower tidal volumes as compared with conventional tidal volumes for patients without acute lung injury: a preventive randomized controlled trial

**DOI:** 10.1186/cc8230

**Published:** 2010-01-07

**Authors:** Rogier M Determann, Annick Royakkers, Esther K Wolthuis, Alexander P Vlaar, Goda Choi, Frederique Paulus, Jorrit-Jan Hofstra, Mart J de Graaff, Johanna C Korevaar, Marcus J Schultz

**Affiliations:** 1 Department of Intensive Care Medicine, Academic Medical Center, Meibergdreef 9, 1105 AZ, Amsterdam, The Netherlands; 2 Department of Internal Medicine, Academic Medical Center, Meibergdreef 9, 1105 AZ, Amsterdam, The Netherlands; 3 Department of Intensive Care Medicine, Tergooi Hospitals, Rijksstraatweg 1, 1261 AN, Blaricum, The Netherlands; 4 Department of Anesthesiology, Tergooi Hospitals, Rijksstraatweg 1, 1261 AN, Blaricum, The Netherlands; 5 Department of Anesthesiology, Academic Medical Center, Meibergdreef 9, 1105 AZ, Amsterdam, The Netherlands; 6 Department of Epidemiology and Biostatistics, Academic Medical Center, Meibergdreef 9, 1105 AZ, Amsterdam, The Netherlands; 7 Laboratory of Experimental Intensive Care and Anesthesiology (L·E·I·C·A), Academic Medical Center, Meibergdreef 9, 1105 AZ, Amsterdam, The Netherlands

## Abstract

**Introduction:**

Recent cohort studies have identified the use of large tidal volumes as a major risk factor for development of lung injury in mechanically ventilated patients without acute lung injury (ALI). We compared the effect of conventional with lower tidal volumes on pulmonary inflammation and development of lung injury in critically ill patients without ALI at the onset of mechanical ventilation.

**Methods:**

We performed a randomized controlled nonblinded preventive trial comparing mechanical ventilation with tidal volumes of 10 ml *versus *6 ml per kilogram of predicted body weight in critically ill patients without ALI at the onset of mechanical ventilation. The primary end point was cytokine levels in bronchoalveolar lavage fluid and plasma during mechanical ventilation. The secondary end point was the development of lung injury, as determined by consensus criteria for ALI, duration of mechanical ventilation, and mortality.

**Results:**

One hundred fifty patients (74 conventional *versus *76 lower tidal volume) were enrolled and analyzed. No differences were observed in lavage fluid cytokine levels at baseline between the randomization groups. Plasma interleukin-6 (IL-6) levels decreased significantly more strongly in the lower-tidal-volume group ((from 51 (20 to 182) ng/ml to 11 (5 to 20) ng/ml *versus *50 (21 to 122) ng/ml to 21 (20 to 77) ng/ml; *P *= 0.01)). The trial was stopped prematurely for safety reasons because the development of lung injury was higher in the conventional tidal-volume group as compared with the lower tidal-volume group (13.5% *versus *2.6%; *P *= 0.01). Univariate analysis showed statistical relations between baseline lung-injury score, randomization group, level of positive end-expiratory pressure (PEEP), the number of transfused blood products, the presence of a risk factor for ALI, and baseline IL-6 lavage fluid levels and the development of lung injury. Multivariate analysis revealed the randomization group and the level of PEEP as independent predictors of the development of lung injury.

**Conclusions:**

Mechanical ventilation with conventional tidal volumes is associated with sustained cytokine production, as measured in plasma. Our data suggest that mechanical ventilation with conventional tidal volumes contributes to the development of lung injury in patients without ALI at the onset of mechanical ventilation.

**Trial registration:**

ISRCTN82533884

## Introduction

Mechanical ventilation is a life-saving strategy in patients with acute respiratory failure. Nevertheless, unequivocal evidence from both experimental and clinical studies indicates that mechanical ventilation has the potential to aggravate lung injury [1-3]. Data from three randomized controlled clinical trials confirmed the existence of ventilator-associated lung injury in patients with acute lung injury (ALI) or its more-severe form, acute respiratory distress syndrome (ARDS), by showing reduced morbidity and mortality in the lower tidal-volume arm [4-6]. As a result of these studies, current guidelines now clearly support the use of lower tidal volume in patients with ALI/ARDS [7]. In contrast, little evidence supports the use of lower tidal volume in critically ill patients without ALI/ARDS, partly because of a lack of randomized controlled trial evidence on the best ventilator strategies in these patients [8].

Pneumonia, aspiration, sepsis, trauma, shock, and multiple blood transfusions are well-described risk factors for ALI/ARDS [9]. Animal studies demonstrate that mechanical ventilation with conventional tidal volume not only may aggravate, but also may initiate lung injury [1,2]. The role of tidal-volume size as a contributor to the development of lung injury in humans is, however, less clear. One study on reduced tidal volume and pressure settings did not show a reduction in mortality but suggested more side effects of lower tidal-volume ventilation in patients at high risk for ALI/ARDS [10]. Conversely, pulmonary procoagulant changes and increased systemic cytokine production were observed in patients without preexisting lung injury receiving conventional-tidal-volume mechanical ventilation during surgery [11,12].

Other studies have challenged these findings [13,14]. Results from cohort studies suggest that mechanical ventilation with conventional tidal volumes may cause or contribute to development of lung injury in critically ill patients who did not have ALI/ARDS at the onset of mechanical ventilation [15,16]. The inconclusive results from the studies in surgical patients may arise from the fact that these patients were ventilated for only a short period, whereas the patients of the larger cohort studies were critically ill patients who had been ventilated for a longer period.

As ALI/ARDS is characterized by a profound production of inflammatory mediators, it might be expected that if conventional tidal volumes contribute to development of lung injury, the injury also may be associated with increased production of cytokines. We therefore conducted a trial to determine whether mechanical ventilation with conventional or lower tidal volume would be associated with different cytokine patterns in the lungs and the plasma of critically ill patients without ALI at onset of mechanical ventilation. Secondary end points were development of lung injury, duration of mechanical ventilation, and mortality.

## Materials and methods

### Participants

From January 2005 until December 2007 patients were recruited in the intensive care departments of one academic and one regional teaching hospital in the Netherlands. The academic ICU is a 28-bed "closed format" department where medical/surgical patients (including neurosurgery/neurology, cardiothoracic surgery, and cardiology patients) were under the direct care of the ICU team. The ICU team comprised 10 full-time ICU physicians, eight subspecialty fellows, 12 residents, and occasionally one intern. The regional teaching ICU is an eight-bed "open format" department with medical/surgical patients (not including neurosurgery and cardiothoracic surgery patients). The ICU team comprised three full-time ICU physicians, five physicians who participate in evening and night shifts, and one resident. The two ICUs had similar standards of practice in terms of mechanical-ventilation and sedation protocols.

Patients were eligible for the study if they did not meet the consensus criteria for ALI/ARDS [17] and needed mechanical ventilation for an anticipated duration of more than 72 hours. Patients had to be randomized less than 36 hours after the onset of mechanical ventilation. Exclusion criteria were age younger than 18 years, participation in other clinical trials, pregnancy, increased uncontrollable intracranial pressure, chronic obstructive pulmonary disease (defined as a forced expiratory volume in 1 second to a forced vital capacity ratio less than 0.64 and daily medication), restrictive pulmonary disease (evidence of chronic interstitial infiltration on chest radiograph), use of immunosuppressive agents (100 mg hydrocortisone per day was allowed), pulmonary thromboembolism, previous pneumectomy or lobectomy, and previous randomization in this study. Randomization was performed by using sealed opaque envelopes in blocks of 50 patients. Each study center had its own randomization block. The protocol was approved by the medical ethics committees of both hospitals, and written informed consent was obtained from the patient or closest relatives before entry in the study. All procedures were done in compliance with the Helsinki declaration.

### Interventions

The volume-controlled mode was used for mechanical ventilation. To calculate tidal volume, predicted body weight was used, as described [4]. The target tidal volume in the conventional group was 10 ml/kg of predicted body weight, which was routine practice at the time of the conduct of the study. Patients from the intervention group were ventilated at tidal volumes of 6 ml/kg of predicted body weight. In case patients were randomized to 6 ml/kg, the attending physician was allowed to increase tidal-volume size to 7 to 8 ml/kg if patients had severe dyspnea, as identified by increased respiratory rate (more than 35 to 40 breaths per minute) accompanied by increasing levels of discomfort (with or without need for more sedation). Levels of PEEP were set, together with the level of inspired oxygen (FiO_2_) depending of the PaO_2 _according to a local protocol.

The ventilator was routinely (3 times/day) switched to the pressure support mode. If the pressure support mode was tolerated, this mode was used for further mechanical ventilation. Toleration of pressure support mode was assessed at the discretion of the attending physician. The pressure support was adjusted to reach the target tidal volumes. In case the attending physician preferred pressure-support ventilation in a patient randomized to the lower-tidal-volume group, and the applied tidal volume exceeded the target tidal volume because of high levels of pressure support, then this was accepted. Such patients were kept in their original randomization group in the statistical analyses.

As soon as patients were ready to be weaned from the ventilator, the pressure-support level had to be lowered stepwise to 5 cm H_2_O within 24 hours. If this was not possible because of severe dyspnea, then the pressure support had to be increased to maintain tidal-volume size based on randomization group. Attending physicians decided to extubate the patient, based on general extubation criteria (that is, responsive and cooperative, adequate oxygenation with FiO_2 _of 40% or less, hemodynamically stable, no uncontrolled arrhythmia, and having a rectal temperature greater than 36.0). If a patient had been weaned from the ventilator but was reintubated for additional mechanical ventilation within 28 days, the same tidal-volume protocol was resumed.

Lung injury was diagnosed if a patient met the consensus criteria [17]. If it was diagnosed by the attending physician, the local protocol mandated mechanical ventilation with a tidal volume of 6 ml/kg in a pressure-controlled mode for the remaining ventilation period.

### Objective and outcomes

The primary outcome was cytokine levels in blindly obtained bronchoalveolar lavage fluid and plasma. Development of lung injury (according to consensus criteria for ALI/ARDS) [17], duration of mechanical ventilation, and mortality were secondary outcomes.

### Data collection

Demographic data, ventilation parameters, and clinical and radiologic data were recorded immediately after the ventilator settings were changed on day 0. Each second day, ventilator settings, blood-gas parameters, radiographic data, and medication use were recorded until the patient was weaned from the ventilator. The oxygenation index was calculated as described earlier [18]. Mean airway pressure was measured with the ventilator. The lung-injury score (LIS) was calculated. On the day of enrollment and each second day until the patient was weaned from the ventilator, a bronchoalveolar minilavage was performed for the measurement of levels of tumor necrosis factor α (TNF-α), interleukin-1β (IL-1β), and interleukin-6 (IL-6). Simultaneously, blood samples were drawn from an indwelling arterial catheter for IL-6 measurements. Minilavage was performed as described previously [19]. The recovered fluid was centrifuged at 1,500 *g *for 10 minutes at 4°C. The supernatant was collected and stored at -80°C until measurements were performed. All markers were measured with an enzyme-linked immunoassay (Sanquin, Amsterdam, The Netherlands).

### Definitions

Sepsis was defined by the Bone criteria [20]. Septic shock was present in cases of persisted hypotension (mean, less than 60 mm Hg) despite fluid resuscitation or vasopressor use [20]. Pneumonia was diagnosed from new infiltrates on chest radiograph together with clinical signs of infection and positive sputum culture with no other explanation for the symptoms [21]. Chronic alcohol abuse was defined as a previously established diagnosis of chronic alcoholism, a prior admission for alcohol detoxification, or alcohol withdrawal [22].

### Sample size

The power calculation was based on a previous study on ventilator-associated lung injury [23]. In this study, bronchoalveolar lavage fluid levels of IL-6 increased by ± 20% in ALI/ARDS patients ventilated with a conventional regimen and decreased by ± 20% in patients ventilated with a protective regimen. Based on these differences and expected baseline IL-6 levels of 250 pg/ml [19], we calculated that to detect a difference in changes from baseline between groups of 100 pg/ml, with a two-sided significance level of 0.05 and a power of 80%, 49 patients had to be included in each group. As we studied patients without ALI/ARDS, we chose to study twice as many patients, resulting in a total of 200 patients.

### Lung injury diagnosis for interim analysis

For reasons of safety, interim analyses on the development of lung injury were conducted after the inclusion of 100 and 150 patients. For this, all chest radiographs were reviewed by two independent physicians who were blinded to all clinical parameters and randomization groups. Any new or worsening abnormality was scored. Chest radiographs showing new or worsening abnormalities were selected for further review. During the review process, they had access to PaO_2_/FiO_2 _(P/F), echocardiography, and fluid-balance data, pulmonary capillary wedge pressures (if measured), and the admission diagnosis. Both physicians were familiar with the consensus criteria for ALI/ARDS [17]. In case of disagreement, consensus had to be obtained while reviewing the patient together.

### Statistical analysis

Data are presented as mean with standard deviation for parametric data or as medians with interquartile range (IQR) for nonparametric data. Baseline comparisons between groups were made with the Student *t *test, Mann-Whitney *U *test, χ^2 ^test, or Fisher Exact test where appropriate. The Mann-Whitney *U *test was used to compare baseline levels of cytokines between groups. To study the primary outcome, a linear mixed model was constructed on cytokine levels, adding time and randomization group as factors in the model. In this model, the interaction between time and randomization group was used to study differences over time between groups. If the residuals were not normally distributed in linear mixed-model analyses, the data were transformed to the natural logarithm of the original data. The relation between cytokine levels and development of ALI/ARDS was studied with a multivariate logistic regression analysis. For the secondary outcome, development of ALI/ARDS was studied with the χ^2 ^test. To show the incidence of ALI/ARDS over time, a Kaplan-Meier curve was constructed, and the log-rank test was used to calculate differences between groups.

To study the effect of tidal volumes while correcting for risk factors for ALI, a multivariate logistic regression analysis was performed. Variables with a *P *value < 0.10 in univariate analysis were considered for a multivariate model. If collinearity between variables was found, then the weaker variables were removed from the multivariate model. A backward elimination method was used for the final model.

A two-tailed *P *value < 0.05 was considered to be statistically significant. Data were analyzed by using SPSS, version 14.02 (SPSS Inc., Chicago, IL).

## Results

### Patients

**Figure 1:**
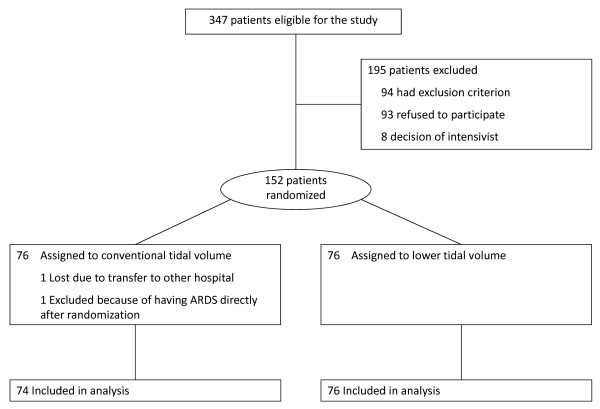
**Flow diagram summarizing inclusion, allocation, and analysis**. 347 patients were eligible for the study; 94 patients were excluded because of participation in another clinical trial (*n* = 49), use of immunosuppressive agents (*n* = 22), chronic obstructive pulmonary disease (*n* = 11), prior pneumectomy or lobectomy (*n* = 5), interstitial lung disease (*n* = 4), and pulmonary thromboembolism (*n* = 3); 93 patients refused informed consent, and in eight patients, participation in the trial was denied by the attending physician.

A flow diagram summarizing patient inclusion, allocation, and analysis is given in Figure 1. At the second interim analysis, after 150 patients were included, the trial was stopped because more patients had developed lung injury in the conventional tidal-volume group as compared with the lower tidal-volume group ((10 patients (13.5%) *versus *two patients (2.6%); *P *= 0.01)). Demographics and admission diagnoses are shown in Tables 1 and 2. Study groups were well balanced with respect to the number of patients with P/F less than 40 kPa and unilateral chest radiographs abnormalities, the number of patients with bilateral chest radiographs abnormalities but P/F more than 40 kPa, and risk factors for ALI/ARDS. Patients randomized to the lower-tidal-volume group, however, tended to be older, and more patients were chronic smokers.

**Table 1:** Demographic data

	Conventional tidal volume group (*n* = 74)	Lower tidal volume group (*n* = 76)	*P *value
Age (years, mean ± SD)	58 (± 17)	63 (± 15)	0.06
Male sex (*n*, %)	50 (68%)	49 (64%)	0.69
Mechanical ventilation time before randomization (hours, mean ± SD)	20 (± 9)	18 (± 9)	0.25
Tidal volume before randomization (ml/kg ideal body weight, mean ± SD)	8.2 (± 0.4)	8.4 (± 0.6)	0.31
APACHE II score (mean ± SD)	20 (± 8)	21 (± 7)	0.93
SOFA score (mean ± SD)	8 (± 4)	7 (± 3)	0.19
LIS (mean ± SD)	1.2 (± 0.6)	1.3 (± 0.6)	0.08
P/F (mean ± SD)	40.0 (± 8.9)	36.0 (± 11.4)	0.14
ALI/ARDS consensus criteria			0.91
PF >40 and normal CXR	17	17	
PF >40 and abnormal CXR	6	6	
PF <40 and normal CXR	33	34	
PF <40 and unilateral CXR abnormality	18	19	
PF <40 and bilateral abnormality with heart failure	0	1	
Underlying ALI risk factors			0.60
Sepsis	7	4	
Shock	6	9	
Pneumonia	1	3	
Glasgow Coma Scale ≤ 8	19	13	
Trauma	12	10	
Other	2	2	
Blood transfusion	30 (41%)	36 (47%)	0.40
Any blood products (median, IQR)	0 (0-15)	0 (0-4)	0.52
Packed red cells (median, IQR)	0 (0-2)	0 (0-0)	0.25
Filtered red cells (median, IQR)	0 (0-2)	0 (0-1)	0.08
Platelets (median, IQR)	0 (0-0)	0 (0-0)	0.13
Fresh frozen plasma (median, IQR)	0 (0-3)	0 (0-2)	0.08
Chronic alcohol abuse	6 (8%)	5 (7%)	0.72
Current smokers	45 (61%)	58 (76%)	0.04

APACHE-II = acute physiology and chronic health evaluation-II; SOFA = sequential organ failure assessment; LIS = lung injury score. PF = PaO_2 _to FiO_2 _ratio. Other ALI risk factors: aspiration pneumonitis, pancreatitis, massive blood transfusion, drug overdose. The number of blood products is expressed as median with interquartile range per patient with at least one product.

**Table 2:** Admission diagnoses

	Conventional tidal volume group (*n* = 74)	Lower tidal volume group (*n* = 76)
Cardiac arrest	22	32
Neurologic disease	24	15
Sepsis	7	4
Pneumonia	1	3
Aspiration	--	1
Trauma	12	10
Pancreatitis	--	1
Medical other	5	5
Cardiopulmonary surgery	1	3
Other surgery	2	2

### Ventilation data

**Figure 2:**
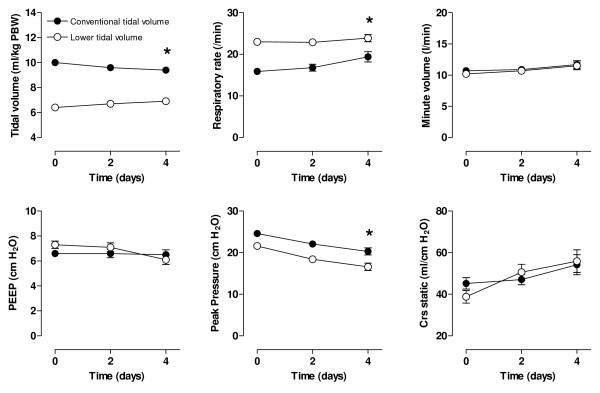
**Serial data on mechanical ventilation parameters of patients ventilated with conventional tidal volume (solid circles) or lower tidal volumes (open circles)**. The number of patients was 74 *versus *76 (conventional *versus *lower tidal volumes), 55 *versus *63, and 34 *versus *34, respectively, at T = 0, T = 2, and T = 4 days. *P < 0.05 (Interaction time × Group).

Ventilator data are presented in Figure 2. Applied tidal volumes were lower in the lower-tidal-volume group as compared with the conventional-tidal-volume group at baseline after randomization (6.4 ± 1.0 ml/kg *versus *10.0 ± 1.0 ml/kg; *P *< 0.001), as was the maximum airway pressure (21.6 ± 7.0 cm H_2_O *versus *24.6 ± 6.7 cm H_2_O; *P *= 0.009). Both remained lower during the study period (Figure 2). Minute ventilation was comparable at baseline and remained comparable during the study period in both study groups. Respiratory rate was higher at baseline and remained higher in the lower-tidal-volume group (*P *< 0.001).

**Figure 3:**
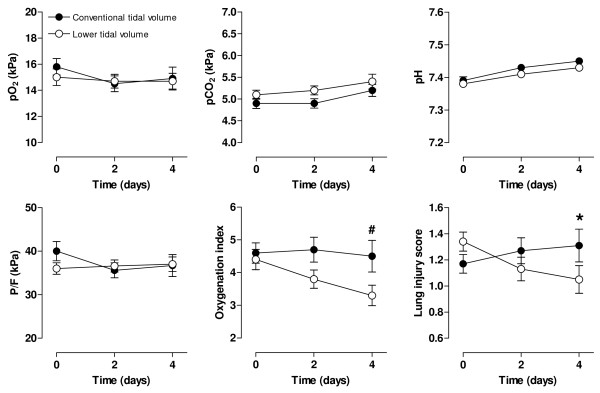
**Serial data on respiratory values and lung-injury score of patients ventilated with conventional tidal volume (solid circles) or lower tidal volumes (open circles)**. PaO_2 _partial pressure of arterial oxygen; PaCO_2 _partial pressure of arterial carbon dioxide; PF = ratio of PaO_2 _to fraction of inspired oxygen; LIS lung injury score. The number of patients was 74 *versus *76 (conventional *versus *lower tidal volumes), 55 *versus *63, and 34 *versus *34, respectively, at T = 0, T = 2, and T = 4 days. *P < 0.05; ^#^*P *= 0.06 (Interaction time × Group).

No differences were observed in the static compliance (Figure 2), blood-gas analysis data, and P/F between the study groups (Figure 3). However, a trend toward a difference in the oxygenation index after 4 days was noted between study groups (*P *= 0.06), and LIS significantly increased after 4 days in the conventional-tidal-volume, whereas it decreased in the lower-tidal-volume group (linear mixed models, interaction time, and group, *P *= 0.003).

### Cytokine levels

**Figure 4:**
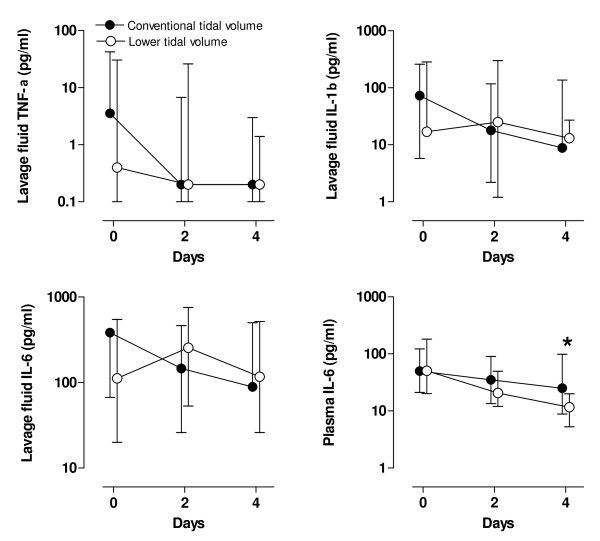
**Serial data on cytokine levels in bronchoalveolar lavage fluid of patients ventilated with conventional tidal volume (solid circles) or lower tidal volumes (open circles)**. TNF-α = tumor necrosis factor-α; IL-1β = interleukin-1β; IL-6 = interleukin-6. The number of patients was 74 *versus *76 (conventional *versus *lower tidal volumes), 55 *versus *63, and 34 *versus *34, respectively, at T = 0, T = 2, and T = 4 days. *P < 0.05; ^#^*P *= 0.06 (Interaction time × Group).

Baseline lavage-fluid levels of TNF-α and IL-1β were comparable in both study groups; baseline lavage-fluid levels of IL-6 were higher in the conventional group, although statistical significance was not reached ((384 (67 to 1,136) pg/ml *versus *112 (20 to 548) pg/ml; *P *= 0.07)) (Figure 4). Lavage-fluid levels of cytokines remained comparable over time in both study groups. Baseline plasma IL-6 levels were comparable in both study groups ((50 (21 to 122) ng/ml *versus *51 (20 to 182) ng/ml in the conventional- and lower-tidal-volume groups, respectively; *P *= 0.74)). In the conventional-tidal-volume group, plasma IL-6 levels decreased after 4 days ((21 (9 to 99) ng/ml)), but the decrease over time was more pronounced in the lower-tidal-volume group ((11 (5 to 20) ng/ml; *P *= 0.01)).

**Figure 5:**
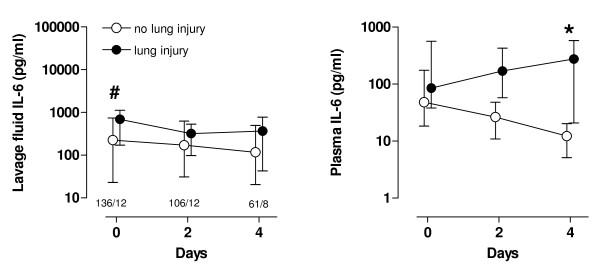
**Serial data on cytokine levels in lavage fluid and plasma of patients in whom ALI/ARDS developed (solid circles) and of patients in whom it did not (open circles)**. The number of patients was 136 *versus *12 (no lung injury *versus *lung injury), 106 *versus *12, and 61 *versus *8, respectively, at T = 0, T = 2, and T = 4 days. *P < 0.05.

As compared with patients in whom lung injury did not develop, patients in whom lung injury did develop had significantly higher baseline lavage-fluid levels of IL-6 ((593 (148 to 1,321] pg/ml *versus *226 (23 to 765) pg/ml; *P *= 0.04) (Figure 5). Lavage IL-6 levels remained elevated after 4 days. Although baseline plasma levels of IL-6 were comparable between patients in whom lung injury did and did not develop, levels increased after 4 days in patients in whom lung injury developed (Figure 5; *P *= 0.01).

### Clinical outcome data

**Figure 6:**
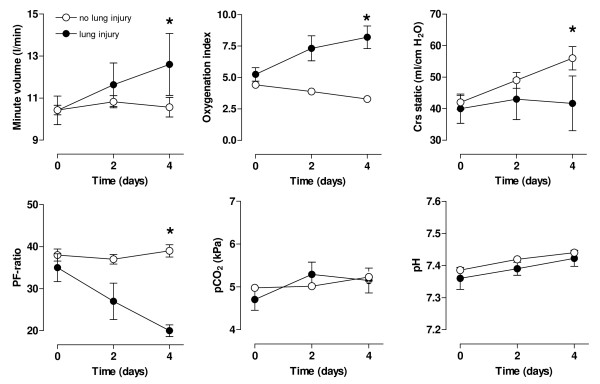
**Serial data on minute volume (left graph), oxygenation index (middle graph), and lung compliance (right graph) in patients in whom lung injury developed (solid circles) and in patients in whom it did not (open circles)**. The number of patients was 136 *versus *12 (no ALI/ARDS *versus *ALI/ARDS), 106 *versus *12, and 61 *versus *eight, respectively, at T = 0, T = 2, and T = 4 days. *P < 0.05.

Twenty-five patients had new or worsening abnormalities on their chest radiographs; 12 patients met the consensus criteria for ALI/ARDS after 1.9 ± 1.1 days. Ten of these were randomized to conventional tidal volume, and two, to lower tidal volume mechanical ventilation (*P *= 0.01; χ^2 ^test), leading to a relative risk of 5.1 (95% CI, 1.2 to 22.6) for developing lung injury. Patients in whom lung injury developed diverged from patients in whom lung injury did not develop, with respect to minute ventilation, LIS, and static compliance (Figure 6). P/F and oxygenation index changed significantly after 4 days in patients in whom lung injury developed. Underlying risk factors in ALI/ARDS patients were sepsis (*n* = 4), shock (*n* = 1), trauma (*n* = 1), drug overdose (*n* = 1), and multiple blood transfusions (*n* = 1) in the conventional-tidal-volume group, and pneumonia (*n* = 1) and shock (*n* = 1) in the lower-tidal-volume group.

**Figure 7:**
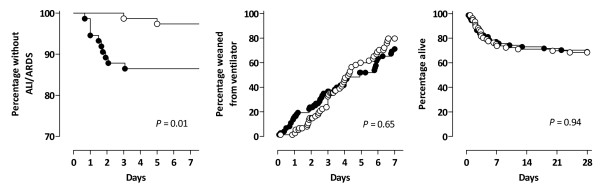
**Kaplan-Meier curve of incidence of acute lung injury (left graph), percentage of patients weaned from ventilator (middle graph), and mortality (right graph) in patients mechanically ventilated with conventional tidal volume (solid circles) or lower tidal volumes (open circles)**.

After 7 days, 13 (25%) of the surviving patients from the conventional-tidal-volume group and nine (17%) from the lower-tidal-volume group were still on the ventilator (*P *= 0.31). After 28 days, the number of ventilator-free days was not different between groups: 24.0 (20.7 to 26.8) days in the conventional-tidal-volume group and 24.0 (21.5 to 25.5) days in the lower-tidal-volume group (*P *= 0.88). After 28 days, 23 (31%) patients from the conventional-tidal-volume group and 24 (32%) patients from the lower-tidal-volume group had died (*P *= 0.94). The Kaplan-Meier curves are shown in Figure 7.

The number of days on which sedatives were used was not significantly different between study groups. In the conventional-tidal-volume group, sedation was used for 1.9 ± 3.5 days *versus *1.7 ± 2.2 days in the lower-tidal-volume group (*P *= 0.69). Neuromuscular blocking drugs were used only to facilitate tracheal intubation. The number of days on which vasopressors or inotropic agents were used also was comparable in both study groups (1.8 ± 3.5 days *versus *1.5 ± 1.9 days, in conventional- and lower-tidal-volume groups, respectively; *P *= 0.64).

### Univariate and multivariate analysis

Univariate analysis showed statistical relations between baseline LIS, randomization group, level of PEEP, the number of blood products, ALI/ARDS risk factor, and the baseline IL-6 lavage-fluid level with development of lung injury. Multivariate analysis revealed the randomization group and level of PEEP as independent predictors of lung injury in this study (Table 3).

**Table 3:** Univariate and multivariate logistic regression analysis of risk factors associated with development of acute lung injury

	Lung injury (*n* = 12)	No lung injury (*n* = 138)	*P *value univariate analysis	*P *value multivariate analysis
Age (years)	63.5 (54.3-78.3)	63.0 (48.8-74.0)	0.41	-
Male gender (*n*, %)	9 (75%)	90 (65%)	0.49	-
APACHE-II score	19 (15-23)	20 (15-27)	0.82	-
LIS	1.5 (1.25-2.0)	1.25 (0.75-1.75)	0.03	-
ALI-risk factor (*n*, %)	10 (83%)	82 (59%)	0.10	0.14
Number of transfused blood products (median, IQR)	0 (0-13)	0 (0-2)	0.01	-
Oxygenation Index	5.2 (4.3-6.8)	3.9 (2.7-5.5)	0.04	-
PaO_2_/FiO_2_	34 (26-41)	(35 (28-45)	0.46	-
Conventional-tidal-volume group	10 (83%)	64 (46%)	0.01	0.007
PEEP level (cm H_2_O)	10 (8-12)	5 (5-9)	0.001	0.001
IL-6 level in lavage fluid (median with IQR, pg/ml)	592 (148-1,321)	226 (23-765)	0.04	-
IL-6 level in plasma (pg/ml)	79 (25-565)	48 (19-145)	0.13	-
TNF-α level in lavage fluid (median with IQR, pg/ml)	7.2 (1.0-121)	1.3 (0-30.6)	0.11	-
IL-1β level in lavage fluid (median with IQR, pg/ml)	9.0 (0.0-96.7)	42.4 (4.2-281)	0.32	-

APACHE-II = acute physiology and chronic health evaluation-II; LIS = lung injury score. Data are expressed as medians with interquartile range or as number with percentage. Underlying risk factors in patients in whom lung injury developed were sepsis (*n* = 4), shock (*n* = 1), trauma (*n* = 1), drug overdose (*n* = 1), and multiple blood transfusions (*n* = 1) in the conventional-tidal-volume group, and pneumonia (*n* = 1) and shock (*n* = 1) in the lower-tidal-volume group.

## Discussion

ALI/ARDS is rarely present at the time of hospital admission but develops over a period of hours to days in patients with predisposing conditions, such as trauma, shock or sepsis, and associated interventions, including mechanical ventilation [24]. Therefore, ALI/ARDS may be viewed as a potentially preventable complication. Implementation of prevention strategies, such as lung-protective mechanical ventilation with lower tidal volumes lead to a significant decrease in ALI/ARDS and the mortality of mechanically ventilated patients [25]. Although both groups in the present study had a comparable prevalence of risk factors for ALI/ARDS, mechanical ventilation with conventional tidal volumes was associated with a delayed decrease in plasma IL-6 levels and an increased frequency of lung injury after the initiation of mechanical ventilation. The benefit of the use of lower tidal volumes occurred without the need for additional sedation or vasopressor use and was not associated with altered requirements for higher PEEP or additional FiO_2_.

Conventional mechanical ventilation was accompanied by an altered plasma cytokine profile but not an altered pulmonary cytokine profile. We found plasma IL-6 levels to decrease over time in both groups. The decrease was, however, more pronounced in patients ventilated with lower tidal volumes. This was not accompanied by different cytokine profiles in the lavage fluids. This is in contrast with findings of earlier studies in patients undergoing elective surgery. In a recent study, procoagulant changes in lavage fluid of patients with healthy lungs were observed after 5 h of mechanical ventilation with large tidal volumes [11]. Another recent trial showed increased blood cytokine levels in surgical patients ventilated with conventional tidal volumes compared with those in patients ventilated with lower tidal volumes [12]. The increase in lung-injury score in the conventional-tidal-volume group may not have been reflected by increases in cytokine levels because of a different timing of lavage, deteriorations in P/F, and changes on chest radiographs. A lavage was performed each second day, but the chest radiographs could be made daily, and blood-gas analyses were routinely performed at least 4 times per day. Moreover, in seven patients, the attending physician reduced the tidal volume size to 6 ml/kg after the development of lung injury. The study protocol allowed only lung lavage every second day. Lavage procedures were not always performed on the moment of ALI/ARDS diagnosis and before tidal volumes were reduced.

Although we did not observe a general increase in cytokine levels, the mechanism by which mechanical ventilation with conventional tidal volumes leads to full-blown ALI/ARDS in critically ill patients may be as follows. A second-hit model theory can be suggested as a mechanism by which mechanical ventilation may lead to ALI/ARDS. The patients in whom lung injury developed in our study had increased IL-6 levels in their lavage fluid, a higher level of PEEP, and a worse oxygenation index. Although the IL-6 level and oxygenation index were not independent predictors in multivariate analysis, it does show that these patients had some pulmonary inflammation at baseline. It may be speculated that patients with a certain level of inflammation are the patients at risk for ventilator-induced lung injury. Moreover, the baseline level of PEEP was significantly associated with development of lung injury. Larger studies also showed that next to tidal-volume size, risk factors for ALI/ARDS, level of PEEP, and P/F are significant predictors of ALI/ARDS, which is in line with the second-hit model theory [15,16].

The study was stopped after the second interim analysis. Interim analyses were not planned at first and were not taken into account on the calculation of sample size, which is a limitation of our study. Although development of lung injury was a secondary end point in our study, the attending physicians of the Academic Medical Center insisted on interim analyses, as they had concerns about the safety of the study. They assumed that the development of lung injury was more frequent with the use of conventional tidal volumes. Therefore, interim analyses were planned halfway and after 150 patients. A stopping boundary was not determined beforehand. As the *P *value was as low as 0.01 on the second interim analysis, the investigators had no other choice than to stop the trial.

The multivariate analysis showed that tidal volume was an independent predictor of ALI/ARDS development, together with the level of PEEP. Our study was not powered to investigate various risk factors for ALI/ARDS separately in a multivariate model. Therefore, the results of this analysis should be taken with caution. As the level of PEEP may simply have been a marker of disease severity, tidal-volume size may be the only risk factor that can be influenced by the attending physician. Risk factor, oxygenation index, number of transfused blood products, and the baseline lavage-fluid IL-6 level were all associated with lung injury in the univariate analysis and tended to have a significant influence in the multivariate model. As lung injury was a secondary outcome, this study was not powered to investigate all these variables, but larger studies showed that these are significant predictors of ALI/ARDS [15,16]. This is in line with the suggested second-hit model described earlier.

Our findings are in line with earlier reports in patients without ALI/ARDS at the onset of mechanical ventilation [15,16,23,24]. The odds ratio of 5.1 for a tidal volume of 10 ml/kg *versus *6 ml/kg in the present study is in line with earlier findings of an odds ratio of 1.3 for each milliliter above 6 ml/kg [15]. Although the incidence of development of lung injury in the conventional-tidal-volume group of our study seems quite high (13.5%), it is still low compared with a comparable cohort of patients without preexisting lung injury in another setting [24]. Of interest, the frequency of ALI/ARDS decreased approximately 65% after implementation of lung-protective measures, resulting in an incidence of ALI/ARDS comparable to that in our study [25].

The reported mortality rates in ALI/ARDS patients are, however, relatively high, even with lower-tidal-volume mechanical ventilation [4]. We did not find differences in either mortality or the number of ventilator-free days, however. First, our study was not powered to these end points. Second, the protocol required that if lung injury developed, tidal volumes were reduced to 6 ml/kg. This may have underestimated the effect of higher tidal volumes.

Although diagnosing ALI/ARDS is susceptible to subjective interpretation [26], the final decision on the development of lung injury was made by two physicians who were independent of the study. Their judgment was supported by differences in LIS, compliance, and pulmonary and plasma IL-6 levels between patients with and without lung-injury development. Nevertheless, although the physicians were experienced, it may still be possible that some patients who were identified as having ALI/ARDS had hydrostatic pulmonary edema or pulmonary infection and that patients identified as not having ALI/ARDS actually had ALI/ARDS. This may have happened in cardiac-arrest patients, as they are prone to have elevated left atrial pressures and cardiogenic pulmonary edema. Furthermore, as this patient group has a poor prognosis, ALI/ARDS may have been overlooked by the attending physicians. However, all patients with respiratory failure in this group were reviewed by the independent intensivists. As the clinical assessment of ALI/ARDS is hampered by the lack of a gold standard [26], the sensitivity and specificity of any scoring system is moderate at best. The same holds true for the clinical assessment of infection and the identification of patients with ventilator-associated pneumonia [27]. Despite these limitations, we observed an incidence of 8% in the whole group, which is in line with an earlier report of 6% in a large international cohort study [16].

## Conclusions

Mechanical ventilation with conventional tidal volumes is associated with sustained cytokine production, as measured in plasma. Our data at least suggest that mechanical ventilation with conventional tidal volumes contributes to development of lung injury in patients without ALI at onset of mechanical ventilation. The use of lower tidal volumes did not affect the sedation needs or vasopressor use and was not associated with altered requirements for higher PEEP or additional FiO_2_. As tidal-volume settings can be determined by physicians, the incidence of this iatrogenic form of lung injury may be reduced. Whether reducing tidal volumes benefits patients with respect to the duration of mechanical ventilation and lower mortality rates remains to be determined in a larger randomized controlled trial.

## Key messages

• Mechanical ventilation with conventional tidal volumes in patients without ALI is associated with sustained cytokine production, as measured in plasma.

• Our data at least suggest that mechanical ventilation with conventional tidal volumes contributes to the development of lung injury in patients without ALI at the onset of mechanical ventilation.

• The use of lower tidal volumes is not associated with higher sedation needs or vasopressor use.

• The use of lower tidal volumes is not associated with requirements for higher PEEP or additional FiO_2_.

• Larger randomized controlled trials are needed to confirm whether reducing tidal volumes benefits patients with respect to shorter duration of mechanical ventilation and lower mortality rates.

## Abbreviations

ALI: acute lung injury; ARDS: acute respiratory distress syndrome; IL-1β: interleukin-1β; IL-6: interleukin-6; LIS: lung injury score; PEEP: positive end-expiratory pressure; TNF-α: tumor necrosis factor-α; SOFA: sequential organ-failure assessment.

## Competing interests

The authors declare that they have no competing interests.

## Authors' contributions

The study was designed by RMD, EKW, LC, and MJS. Acquisition of the data was performed by RMD, AR, EKW, APV, GC, FP, JJH, and MJG. Analysis and interpretation of data was done by RMD, JK, and MJS. The manuscript was drafted by RMD, JK, and MJS. Critical revision of the manuscript for important intellectual content was done by RMD, AR, EKW, AV, GC, FP, JJH, MJG, JK, and MJS. The statistical analysis was done by RMD, JK, and MJS. Final approval of the manuscript was done by RMD, AR, EKW, APV, GC, FP, JJH, MJG, JK, and MJS.
